# Efficacy of rodenticide baits with decreased concentrations of brodifacoum: Validation of the impact of the new EU anticoagulant regulation

**DOI:** 10.1038/s41598-019-53299-8

**Published:** 2019-11-14

**Authors:** Marcela Frankova, Vaclav Stejskal, Radek Aulicky

**Affiliations:** Crop Research Institute, Drnovska 507, CZ-16106 Prague, Czech Republic

**Keywords:** Urban ecology, Animal physiology

## Abstract

Anticoagulants are the most frequently used rodenticides at the global scale. Because of their persistency, bioaccumulation and potential for secondary intoxication, they have faced increasing legislative regulations. Recently, the European Union Regulation (EU) 2016/1179 resulted in the production and application of rodenticides with nearly half dose (<30 ppm) of anticoagulants. However, published data on the biological efficacy of rodenticides with decreased doses are scarce in the EU. Therefore, this work compared the efficacy of the original high-dose (50 ppm) and new low-dose (25 ppm) brodifacoum-based baits in the offspring of wild-caught house mice (*Mus musculus* L.). In the no-choice laboratory feeding tests, 100% animals died in all treated groups and 0% died in the control groups. The achieved time to death did not differ between the original and low-dose baits across both types of feeding trials/regimes. The low-dose baits (25 ppm) were consequently tested under field conditions in two populations showing 95.7% and 99.8% efficacy. The obtained results highlighted the good efficacy of the new baits based on low-dose brodifacoum in non-resistant mouse populations. However, further validation is required regarding the remaining anticoagulant compounds and resistant rodent populations.

## Introduction

Anticoagulants have gradually become essential tools for rodent control since discovering their rodenticide potential when formulated as baits in the 1950s. They are currently used not only for rodent control in agriculture and urban environments^1–3^ but also for the protection of native fauna against invasive rodents, especially in environmental programmes aimed at eradicating rodent pests on islands^4,5^. The major benefit of anticoagulant-based rodenticides is undoubtedly their chronic mode of action; they stop blood coagulation resulting in a fatal haemorrhage, which occurs in 4–12 days. This delayed efficacy prevents rodents from associating the proceeding toxicity with the consumed food, the limiting factor for acute rodenticides. Another advantage of anticoagulant rodenticides is an available treatment via vitamin K in the case of accidental poisoning.

On the other hand, anticoagulants pose an environmental risk via the primary and secondary intoxication of non-target species^6,7^ and are persistent in the environment^8,9^. This is especially the issue of the widely used second-generation anticoagulants, while those of the first-generation pose lower risks to non-target wildlife^10,11^. Thus, anticoagulants are classified as persistent, bioaccumulative and toxic (PBT) substances and do not fulfil the environmental and public health safety criteria required by the European Union (EU) for use as biocidal products (Biocidal Products Regulation (BPR), Regulation (EU) 528/2012). Since anticoagulants meet both the exclusion and substitution criteria of the Regulation, i.e., Article 10 (1), (a) and (e) and Article 5 (1), (c) and (e)^12^, there have been long-term research and development efforts to find alternative, efficient and safe rodenticides. Nevertheless, no sufficient substitution for anticoagulant substances has been found, which results in periodic renewals of approval of these active substances (more recently EU Regulation of 25 July 2017).

Currently, various programmes to mitigate anticoagulant hazards are being established in the EU and elsewhere^13–15^ and are based on environmentally friendly procedures that also include a limitation on continuous use of anticoagulant baits and the introduction of non-toxic baits for rodent monitoring^16^. In addition, the EU Commission recently accepted the recommendation of the European Chemical Agency (ECHA) for the reclassification of currently used anticoagulant rodenticides (Commission Regulation (EU) 2016/1179; applied from 1 March 2018). All rodenticides with a 0.003% dose (30 ppm) of anticoagulant or higher must be labelled by a new hazard symbol “toxic to reproduction” and simultaneously, according to the BPR, such products are forbidden for amateur use. Until the Regulation was applied (1 March 2018), seven of the eight approved anticoagulants (brodifacoum, bromadiolone, chlorophacinone, coumatetralyl, difenacoum, flocoumafen and warfarin) were traditionally used in 0.005% (or more) doses (50 ppm) of anticoagulant in commercial products. The impact of this regulation was the production of rodenticide baits with low doses of anticoagulant (below 30 ppm) to avoid both labelling changes and general public inaccessibility.

These “new” low-dose commercial rodenticides represent an important shift in rodent control practice since their application reduces the ecological impacts and environmental residues of anticoagulant active substances. On the other hand, a low concentration of an active substance in the product may require the consumption of more bait during a single feed and/or visiting the feeding site repeatedly for the ingestion of the lethal dose by a target rodent. Low concentrations may also prolong the period between the initial consumption of the bait and time to death of the individuals. Furthermore, prolonged survival of the targeted individual poses a longer lasting threat for predators to consume the intoxicated animal (secondary intoxication). The pre-lethal effects of anticoagulants on rodent behaviour can cause a shift in general activity to daytime hours, reduced thigmotaxis and general lethargy^17,18^, which make rodents more prone to become prey. Second, there is a spreading phenomenon of genetic resistance to anticoagulants across species and around the world^19^. The use of rodenticides with low concentrations of anticoagulants could accelerate the development and spread of resistance across populations since a nearly two-fold concentration (i.e., 50 ppm) of anticoagulant was widely used in rodenticides during the past four decades.

This work focused on the laboratory and field comparisons of the efficacy of the original high dose and new low-dose brodifacoum-based baits in house mice (*Mus musculus* L.). Brodifacoum is one of the most potent second-generation anticoagulants, which were developed to overcome the problems with resistance to the first-generation anticoagulants in the 1970s. Based on the extensive laboratory and field testing of its efficacy against Norway rats (*Rattus norvegicus* Berkenhout), black rats (*R. rattus* L.) and house mice (i.e., three main rodent pest species), a 50 ppm concentration for broad commercial use was repeatedly recommended^20,21^. Such a high concentration of this potent active ingredient was determined to ensure sufficient control, even in a resistant population of rodents in which a low concentration did not achieve the complete mortality of the targeted animals^21–23^.

The specific goals of this work include a comparison of the efficacy of standard (50 ppm) and low (25 ppm) doses of brodifacoum-based rodenticides (i) in the laboratory and (ii) in the field.

## Methods

### Experimental animals

The strain of wild house mice (*Mus musculus*) originated from an agriculture building in Prague (Czech Republic) in 2017; they were taken from a single small population to minimise genetic variation and sensitivity to anticoagulants. The mice were maintained under standard laboratory conditions (temperature 19–22 °C, 12:12 light cycle). Animals were housed in pairs in standard plastic cages (30 × 15 × 15 cm), and water and food (ST1; Velaz, Ltd., Czech Republic) were provided ad libitum. Captive-bred adult mice of the first generation were used in experiments. Animals were individually housed in the separated experimental wire-mesh cages (26 × 17 × 17 cm).

### Multiple day feeding test

We tested 30 males (mean body weight ± SD = 23.0 ± 3.8 g) and 30 females (mean body weight = 18.4 ± 2.1 g). Mice were randomly assigned to the experimental or control group. Experimental animals (n = 24 males, 24 females; 6 males and 6 females per treatment) were fed continuously a rodenticide bait until all were dead, and the control group animals (n = 6 males, 6 females) were fed a standard diet. Water was provided ad libitum.

Two commercial cereal pellets differing in non-toxic food components were used as rodenticide bait (Norat ATG and Norat H; PelGar, Ltd., Czech Republic). Both baits were tested in two different concentrations of brodifacoum: 50 ppm (standard used in products) and 25 ppm (the lower value of the active substance meeting the requirements of the new EU classification). All baits used were provided by the producer (PelGar).

Mice were examined daily; the remaining food was weighed (including spillage and crumbled food) and replenished. These procedures were repeated daily, and the times to death of the experimental animals were recorded. The experiment was terminated by the death of the last individual of the experimental group.

### Single day feeding test

We tested 28 males (mean body weight ± SD = 20.3 ± 2.2 g) and 28 females (16.2 ± 1.5 g). Mice were randomly assigned to the experimental or control group. Experimental animals (n = 24 males, 24 females; 6 males and 6 females per treatment) were fed a rodenticide bait for 24 hours; the bait was then replaced by a standard diet. The control group (n = 4 males, 4 females) was fed a standard diet. Water was provided ad libitum.

The same treatment (four baits) and experimental procedures as those of the multiple day feeding test were followed.

### Field experiments

The goal of the field experiment was to evaluate the efficacy of two low-dose (25 ppm) baits in agricultural facilities naturally inhabited by wild populations of house mice. Each rodenticide was tested in a different small agricultural facility (approximately 10 × 10 m) includes a room for breeding domestic animals and a separate room for feed storage; both facilities were located in Central Bohemia.

To obtain data on bait efficacy, percentage reduction in rodent activity was used as an estimate of the mortality rate^24^. For this purpose, the consumption of a non-toxic monitoring bait (Hubex Ltd., Czech Republic) was evaluated one week before and again after the treatment. The monitoring baits (54 ± 2 g) were placed in plastic bait stations (Proeko Ltd., Czech Republic; n = 10 for each facility).

During the 3-week treatment period, a defined amount of rodenticide bait (45.6 ± 0.1 and 45.4 ± 0.1 g per bait station for Norat ATG 25 ppm and Norat H 25 ppm, respectively) was laid in bait stations. The baits were monitored, weighed, replenished and replaced with fresh samples in one-week intervals.

### Statistical analysis

To evaluate times to death for the baits (Norat ATG 50 ppm, Norat ATG 25 ppm, Norat H 50 ppm, Norat H 25 ppm) in laboratory feeding experiments, survival analysis comparing multiple samples was applied separately for the multiple and single day feeding tests. Because there were no censored observations, Kruskal-Wallis analysis and multiple comparisons of mean ranks for all groups were subsequently applied to evaluate differences among groups. The Kaplan-Meier survival curves, defined as the probability of surviving in a given time interval, were used for visualisation of the survival data.

Next, the effect of bait exposure period on time to death, i.e., comparison of times to death in multiple versus single day feeding test for each bait, was examined by comparing mean ranks in the Mann-Whitney U test.

Kruskal-Wallis analysis and subsequent multiple comparisons was applied to evaluate food intake (i.e., rodenticide bait for experimental groups and standard diet for control group) in both feeding tests. In multiple day feeding test, individual consumption of food per experiment was expressed as the amount of the consumed food per day and per gram of individual body weight. Next, the 1^st^ day of food consumption (bait or standard diet) and the corresponding brodifacoum dosage, both expressed per gram of individual body weight, were analysed separately for single and multiple day feeding test. The ingested brodifacoum dosage was further compared with the published acute oral median lethal dose (the dose that is lethal for 50% of the animals in the group; LD_50_) of brodifacoum for house mice: LD_50_ = 0.40 mg/kg^22^.

To evaluate the efficacy of rodenticides in the field experiments, the EPPO Standard methodology was used^24^. The mortality rate was estimated by reduction in rodent activity on the basis of the pre- and post-treatment values of consumed monitoring bait for both experiments. The estimated mortality rate = 100 × (pre-treatment index − post-treatment index)/pre-treatment index. Bait consumption during the treatment period were evaluated by the Friedman test.

All calculations were performed using Statistica 12.0 (StatSoft, Inc., Tulsa, OK, USA).

### Ethical approval

All applicable international, national, and/or institutional guidelines for the care and use of animals were followed. The experimental protocol was approved by the Institutional Animal Care and Use Committee of the Ministry of Agriculture of the Czech Republic (permit number 50839/2015-MZE-17214).

## Results

### Laboratory experiments

#### Survival analysis

**Figure 1:**
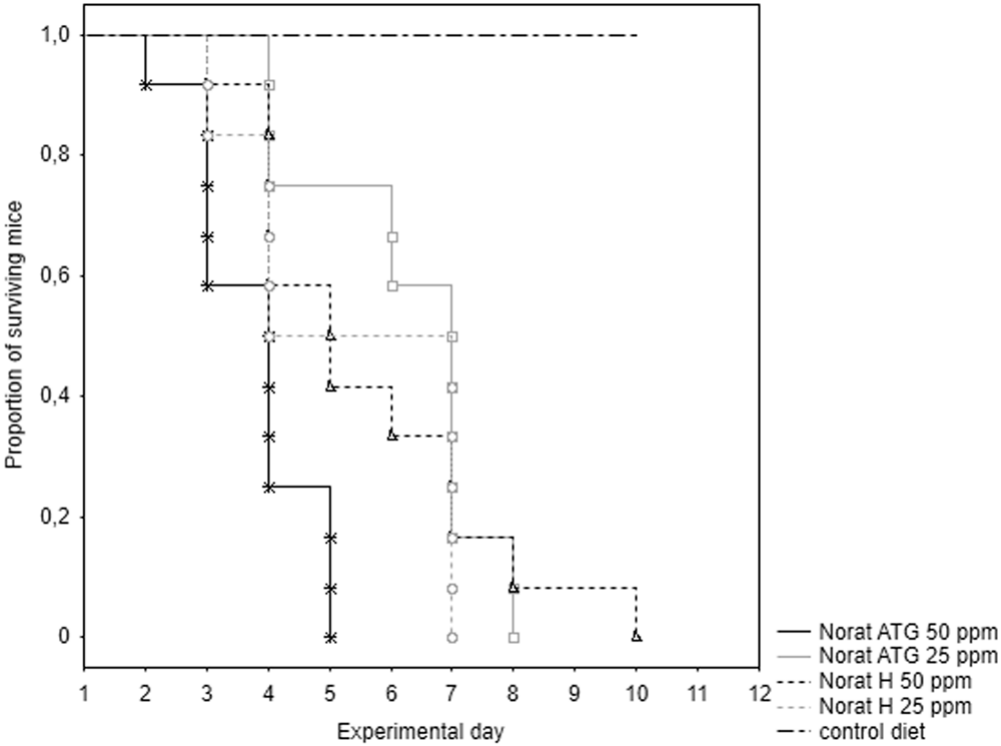
The cumulative survival of house mice (n = 12 for each group) in the multiple day feeding test. Mice were fed two brodifacoum-based baits (Norat ATG, Norat H) at two concentrations: 25 and 50 ppm.

In multiple day feeding test, all experimental mice fed a rodenticide with brodifacoum died and all control mice survived the experiment. Because there were no differences between sexes (all p > 0.05), data for males and females were pooled for further analysis. The survival times differed significantly among experimental groups (chi square = 12.2, p = 0.007). Multiple comparisons of mean ranks revealed that mice fed Norat ATG 50 ppm bait died earlier than those fed Norat ATG 25 ppm bait (p = 0.006; Fig. 1); and the remaining comparisons showed no difference among groups (all p > 0.05; for means, see Table 1).

**Table 1:** Bait intake, brodifacoum dosage and time to death in laboratory feeding no-choice tests. Data are given as the means (±SD).

	Norat ATG 50 ppm	Norat ATG 25 ppm	Norat H 50 ppm	Norat H 25 ppm	control
**Multiple day feeding test**					
1^st^ day food consumption (mg/g)	61.0 (39.6)	274.7 (57.2)	195.2 (47.6)	257.1 (68.9)	180.1 (54.7)
1^st^ day brodifacoum dosage (mg/kg)	3.1 (2.0)	6.9 (1.4)	9.8 (2.4)	6.4 (1.7)	—
Time to death (days)	3.8 (1.0)	6.3 (1.5)	5.6 (2.1)	5.3 (1.8)	—
Average food consumption per day (mg/g)	33.6 (18.7)	161.5 (50.8)	121.8 (45.9)	158.1 (46.2)	177.8 (52.0)
**Single day feeding test**					
1^st^ day food consumption (mg/g)	93.9 (25.1)	301.9 (39.3)	251.6 (23.4)	303.7 (54.2)	205.0 (33.9)
1^st^ day brodifacoum dosage (mg/kg)	4.7 (1.3)	7.5 (1.0)	12.6 (1.2)	7.6 (1.4)	—
Time to death (days)	4.8 (1.7)	4.8 (1.6)	6.4 (1.3)	5.7 (1.3)	—

**Figure 2:**
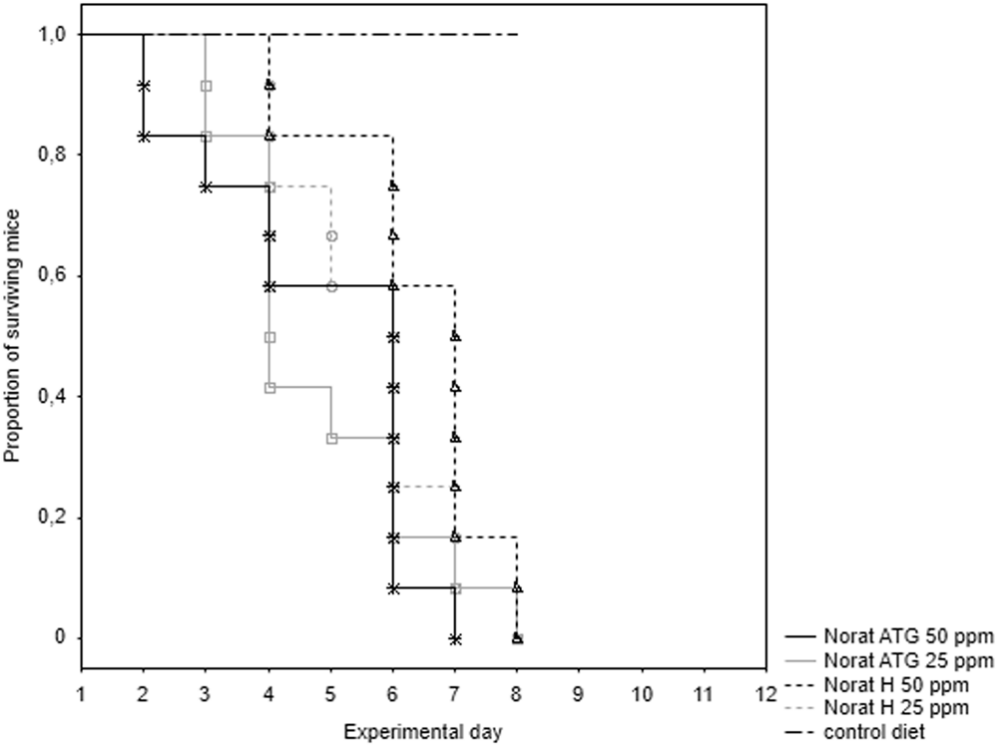
The cumulative survival of house mice (n = 12 for each group) in the single day feeding test. Mice were fed two brodifacoum-based baits (Norat ATG, Norat H) at two concentrations: 25 and 50 ppm.

In single day feeding test, all experimental mice died and all control mice survived the experiment. Because there were no differences between sexes (all p > 0.05), data for males and females were pooled for further analysis. The survival times differed significantly among experimental groups (chi square = 8.6, p = 0.035; Table 1); nevertheless, multiple comparisons of mean ranks showed no significant difference among treated groups (all p > 0.05). The survival curves for the tested baits are presented in Fig. 2.

To evaluate the effect of bait exposure period (multiple versus single day feeding test) on time to death, corresponding times to death for each bait was compared between the feeding tests. The analyses revealed no differences between the feeding tests in all but one comparisons; the only significant difference was found in the case of the Norat ATG 25 ppm bait (Z = 1.99, p = 0.04), where the single day feeding killed mice earlier than the multiple days feeding (mean = 4.8 and 6.3 days for single and multiple day feeding tests, respectively; see also Table 1 for means of all baits).

#### Food intake and brodifacoum dosage

In multiple day feeding test, the average food consumption per experiment (expressed as the amount of the consumed food per day and per gram of individual body weight) differed significantly among groups (H = 33.1, p < 0.001). Multiple comparisons of mean ranks revealed that mice in the Norat ATG 50 ppm group consumed significantly less bait than mice in all other groups (p < 0.001 for Norat ATG 25 ppm, Norat H 25 ppm and control group; p = 0.03 for Norat H 50 ppm group; for means see Table 1).

A similar difference was obtained when only the first day of consumption was analyzed (H = 38.8, p < 0.001). Mice ingested significantly less Norat ATG 50 ppm bait than all other baits (p < 0.001 for Norat ATG 25 ppm, Norat H 25 ppm; p = 0.01 for the Norat H 50 ppm group), and the comparison for the control group remained not significant (p = 0.06). Mean values for each bait and the corresponding dosage of ingested brodifacoum are given in Table 1. Compared with the published acute oral median lethal dose of brodifacoum for house mice (LD_50_ = 0.40 mg/kg)^22^, our experimental mice ingested approximately 8, 17, 24 and 16 times the LD_50_ in the case of Norat ATG 50 ppm, Norat ATG 25 ppm, Norat H 50 ppm and Norat H 25 ppm, respectively.

In single day feeding test, bait consumption differed significantly among groups during the first day (H = 32.3, p < 0.001). The consumption was the lowest in the case of the Norat ATG 50 ppm bait (p < 0.001 for Norat ATG 25 ppm, Norat H 25 ppm; p = 0.03 for the Norat H 50 ppm group). Mean values for each bait and the corresponding dosage of ingested brodifacoum are given in Table 1. Compared with the published acute LD_50_ (0.40 mg/kg)^22^, the experimental mice received approximately 12, 19, 32 and 19 times the dose of LD_50_ in the case of Norat ATG 50 ppm, Norat ATG 25 ppm, Norat H 50 ppm and Norat H 25 ppm, respectively.

### Field experiment

The efficacy of two low-dose baits was evaluated by comparing the consumption of the non-toxic monitoring baits before and after the application of the rodenticide baits. Reduction in rodent activity was used as an estimate of the mortality rate; the values achieved (see Materials and Methods section) 95.7% and 99.8% in the case of Norat ATG 25 ppm and Norat H 25 ppm bait, respectively.

**Figure 3:**
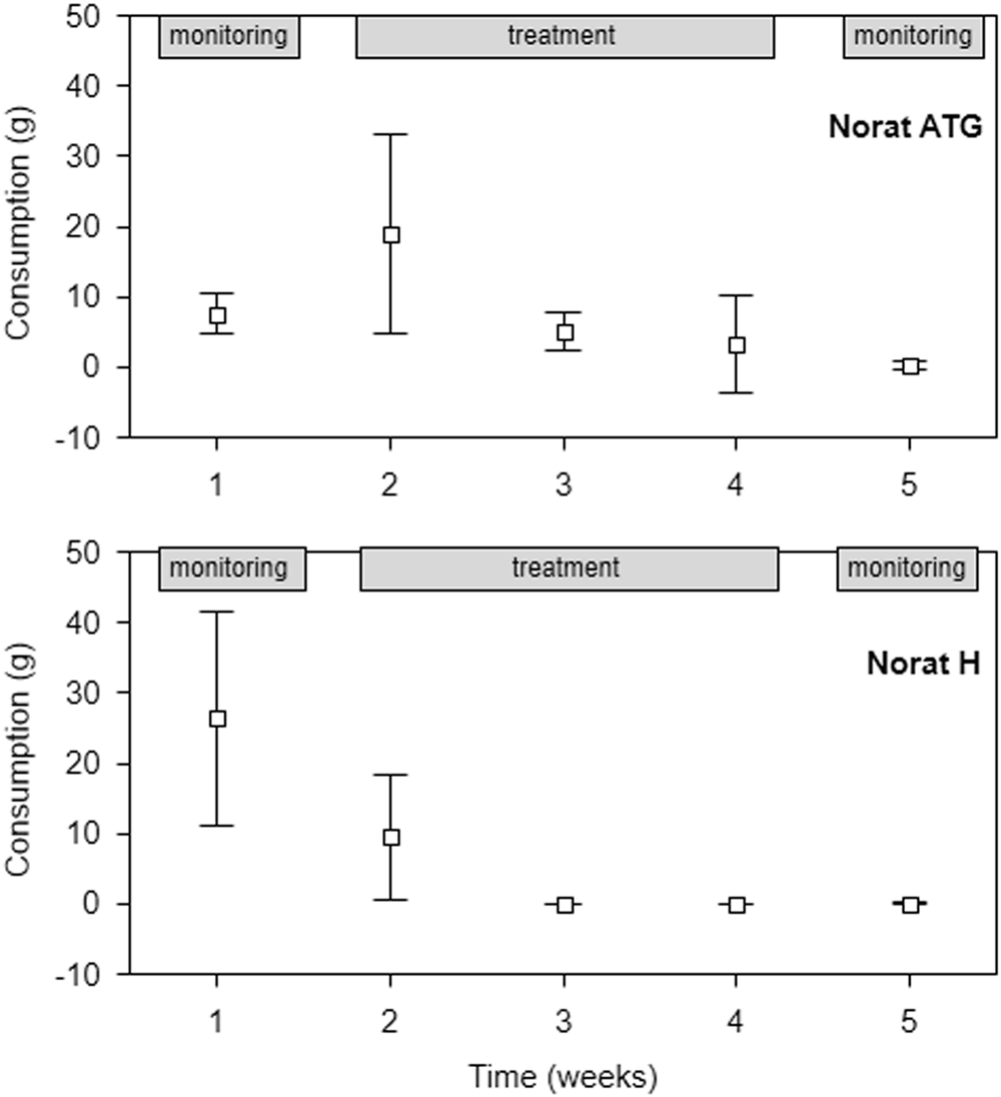
Total bait consumption by house mice in two field experiments. Mice were subjected to the low-dose (25 ppm) baits Norat ATG (upper panel) and Norat H (lower panel) during the treatment phase and to the non-toxic bait during the monitoring phases (displayed in grey boxes). Data are given as the means ± 95% confidence intervals.

Evaluation of the treatment periods revealed differences in bait consumption during the experiment; consumption significantly decreased over time (Norat ATG 25 ppm: chi square = 12.68, p = 0.002; Norat H 25 ppm: chi square = 14.00, p = 0.001; Fig. 3).

## Discussion

The recently approved EU Regulation (No. 2016/1179) resulted in the introduction of “new” anticoagulant-based rodenticide products on the market in March 2018. Compared with the doses in the traditionally used products, these “new” rodenticide baits contained nearly a half-dose of an active ingredient. Because of the limited scientific data on the efficacy of low-dose baits in wild populations of rats and, especially, mice, which are naturally more tolerant to anticoagulants^25,26^, there is a concern about the time period required for the deaths of poisoned rodents. This work addressed the problem regarding the anticoagulant brodifacoum by laboratory and field validation of the original high-dose (50 ppm) and the new low-dose (25 ppm) baits in house mice.

Generally, the results of both laboratory feeding tests showed comparable efficacy for original high-dose and new low-dose baits. The times to death between multiple and single day feeding test did not differ in all but one comparisons of the four tested baits. Interestingly, in this one difference (i.e., Norat ATG 25 ppm bait) even longer time to death in the case of multiple day feeding test was detected (mean = 6.3 and 4.8 days for multiple and single feeding test, respectively). As all tested mice died after the consumption of both 25 and 50 ppm bait within 10 days, we considered them susceptible to brodifacoum. Prolonged survival up to 17–22 days after the continual feeding on brodifacoum-based bait, demonstrating higher tolerance to the rodenticide, was previously documented for 20 and 50 ppm baits^5,27^.

The comparison of survival times between multiple and one day feeding tests revealed that mice died in a similar time period under both treatments. Such a result clearly demonstrates that continual feeding on brodifacoum, i.e., highly potent anticoagulant bait, leads to the ingestion of more multiple doses of the active substance than needed. Such over-dosed individuals create a higher environmental load and risk of secondary intoxication in non-targeted species, i.e., predators and scavengers^7,28,29^. The high palatability of the bait may result in repeated feeding for several days; nevertheless, exclusive feeding on the bait is uncommon under natural conditions, as mice are diffuse and sporadic feeders, regularly visiting many feeding points during their feeding activities^30^. Moreover, we recently showed that anticoagulant bait has the potential to substantially decrease food intake shortly after the initial consumption of the bait, which shortens the activity period of the over-dosed individuals^31^.

The experimental arrangement of this work with unlimited continual bait access was aimed at testing the susceptibility of mice to anticoagulant active substances. During the first day of the experiment, mice ingested 3.1–12.6 mg of brodifacoum per g body weight depending on the amount of consumed bait and brodifacoum dosage (Table 1). Such high amounts exceeded the determined acute oral median lethal dose for house mice (LD_50_ = 0.40 mg/kg)^22^ by 8–31 times. Wheeler *et al*.^5^ examined the efficacy of high brodifacoum dosages (10–15 times LD_50_) in house mice and found that the ingestion of the bait did not result in the complete mortality of the individuals. On the other hand, a simulated field application in another lab experiment showed that repeated or continual bait access ensures complete control of the less susceptible population within 23 days^5^.

One of the key factors in the successful control of rodent pests is the attractivity and palatability of the baits. Although we did not perform the standard food choice tests, our experimental mice consumed different amounts of the tested baits, even in the no-choice tests. There was notably low consumption of the Norat ATG 50 ppm bait in both feeding tests and low, but not significant, consumption of the Norat H 50 ppm bait (Table 1). These results suggested the low palatability of the standard-dose (50 ppm) baits; nevertheless, this issue did not affect the bait efficacy. Similar results on the decreased palatability along with the sufficient efficacy of the 50 ppm (and even 20 ppm) brodifacoum-based bait in house mice were previously published^22^. In any case, the dominant factors for bait palatability are the nature and quality of food ingredients in the baits, which play key roles in the food preference behaviours of rodents^21,32^.

To validate our initial laboratory results, we further tested the efficacy of low-dose baits in the field populations of house mice. Both field trials were evaluated according to the EPPO standards and showed high efficacy of mouse control (95.7% and 99.8% reduction in rodent activity was achieved). A sharp decline in bait consumption was recorded after the first week of application. Such suppression of consumption indicated a reduction in the mouse population, which was further supported by the nearly zero post-treatment consumption of the monitoring bait (see Fig. 3).

Generally, there were extensive efforts to test various dosages of brodifacoum after its introduction to the market in the 1970s and 1980s. The published data present a high degree of efficacy in susceptible and resistant populations^1,20,21,33^. While the high efficacy of even lower dosages (e.g., 10 and 20 ppm) in the susceptible populations was achieved repeatedly^20^ (but see^5,34^), a higher tolerance to 20 or even 50 ppm brodifacoum baits occurred in several warfarin-resistant populations^27,35,36^. This high tolerance to brodifacoum led to the prolonged survival of individuals but not to rodent control failure in general; however, repeated or continual feeding on the bait was necessary^5,27,34,35^. Based on the above-mentioned results, using 50 ppm brodifacoum in baits was repeatedly proposed for effective and comprehensive rodent control, most recently also by ECHA evaluation according to the Biocidal Products Regulation^37^. Nevertheless, the current regulation approach gives priority to reducing the non-target intoxications and environmental load with limited attention to the risk of the spreading of anticoagulant resistance across rodent populations.

In accordance with the BPR, all biocidal products must get authorisation before their introduction to the market. During this authorisation process, the producer provides data on the safety and efficacy of the new product, which are protected by the owner’s claim. There are undoubtedly datasets on the efficacy of the low-dose baits of various active substances, which are unfortunately not published or publicly available. The exceptions are data from studies dealing with rodent eradications on islands where the aerial application of the low-dose brodifacoum baits are commonly used^5,34,38^. Thus, our results could open a discussion on the topic of low-dose anticoagulant baits in the scientific literature, where other related topics are currently highlighted, e.g., resistance and secondary intoxications^2,7,39–41^.

To the best of our knowledge, this is the first study to present laboratory and field data on the efficacy of the new generation of low-dose anticoagulant baits commercially available in the EU in response to the implementation of Regulation (EU) 2016/1179. As we did not find any considerable differences in the efficacy of the original and new low-dose baits, our results are supportive of their application, at least in anticoagulant-sensitive mice strains. Nevertheless, further research is needed to collect data on the efficacy of low-dose baits with other (i.e., less toxic) anticoagulant active substances. As there is a justified concern for low efficacy, at least in some resistant populations (see above), the need for relevant data could be crucial for future regulatory decisions (verify or deny) regarding 50 ppm anticoagulant rodenticides in general.

Future research challenges should also include testing in anticoagulant-resistant populations because only extensive knowledge on anticoagulant resistance for various compounds and populations enables the application of effective rodent control and management with anticoagulant rodenticides.

## Data Availability

The datasets of the study are available from the corresponding author on reasonable request.
